# Nurse-coordinated care improves the achievement of LDL cholesterol targets through more intensive medication titration

**DOI:** 10.1136/openhrt-2017-000607

**Published:** 2017-07-11

**Authors:** Marjolein Snaterse, Harald T Jorstad, Marlies Heiligenberg, Gerben ter Riet, S Matthijs Boekholdt, Wilma Scholte op Reimer, Ron J Peters

**Affiliations:** 1 ACHIEVE Centre for Applied Research, Faculty of Health, Amsterdam University of Applied Sciences, Amsterdam, Netherlands; 2 Department of Cardiology, Academic Medical Center, University of Amsterdam, Amsterdam, Netherlands; 3 Department of General Practice, Academic Medical Center, Amsterdam, Netherlands

**Keywords:** acute coronary syndrome, lipid-lowering medication, LDL-cholesterol target, titration, secondary prevention

## Abstract

**Background:**

Nurse-coordinated care (NCC) improves the achievement of low-density lipoprotein-cholesterol (LDL-C) targets after an acute coronary syndrome (ACS). We hypothesised that NCC improves achievement of LDL-C targets through more intensive medication titration.

**Methods:**

We used data from Randomised Evaluation of Secondary Prevention by Outpatient Nurse Specialists (RESPONSE), a multicentre randomised trial on the efficacy of NCC in 754 ACS patients. Follow-up data were collected at 6 and 12 months. To enable comparison between the various types and dosages of statins, we used the average lipid-lowering potency (ALLP, % LDL-C lowering) as an indicator of lipid-lowering medication intensity.

**Results:**

Most patients in NCC intervention and usual care groups (96%) had started lipid-lowering therapy during the index hospitalisation. At 6 months, titration activities (up or down) were applied in 45% of NCC patients compared with 24% of patients receiving usual care (p<0.001), and a difference was also seen at 12 months follow-up (52% vs 34%, p<0.001). In patients not on LDL-C target at baseline, titration activities at 6 months were recorded in 63% and 30% of NCC and usual care patients respectively (p<0.001), with increased titration activities in both groups at 12 months (69% vs 43%, p<0.001).

**Conclusion:**

NCC is associated with more frequent and intense lipid-lowering medication titration to reach LDL-C targets as compared with usual care alone. Further, merely starting the guideline-recommended dose is insufficient to reach the guideline-recommended LDL-C target level.

**Trial Registration number:**

TC1290 (Netherlands).

**Key questions**

**What is already known about this subject?**

Nurse-coordinated care improves the achievement of low-density lipoprotein-cholesterol (LDL-C) targets after an acute coronary syndrome (ACS).

**What does this study add?**

Nurse-coordinated care is associated with more frequent and intense lipid-lowering medication titration to reach LDL-C targets as compared with usual care alone.

**How might this impact clinical practice?**

Merely starting the guideline-recommended dose is insufficient to reach the guideline-recommended LDL-C target level. Nurse-coordinated care, combined with guideline-based titration recommendations, improves ACS patient outcomes.

## Introduction

Among patients with coronary heart disease (CHD), treatment of risk factors is the cornerstone of secondary prevention.^1^ In the last decade, a substantial increase in antihypertensive and lipid-lowering medication prescriptions has been observed.^2^ Despite a substantial increase in the number of patients receiving guideline-recommended medication, the European Action on Secondary and Primary Prevention by Intervention to Reduce Events (EUROASPIRE) survey showed that up to 3 years after hospitalisation, two-thirds of patients have uncontrolled hypertension, and only half of the patients achieve the guideline-recommended target level for low-density lipoprotein-cholesterol (LDL-C).^3 4^ It has been hypothesised that factors contributing to this suboptimal risk factor control include prescriptions with inadequate dosage, inadequate up-titration of medication, poor adherence of patients to recommended lifestyle changes, poor medication compliance and low standards of follow-up care.^5^

Nurse-coordinated care (NCC) has shown to be a promising strategy to improve secondary prevention, and is currently recommended in the 2016 European prevention guidelines.^1^ In line with this recommendation, we found in a recent systematic review that NCC programmes successfully reduce systolic blood pressure and LDL-C.^6^ However, a clear understanding of how NCC improves achievement of LDL-C targets is still needed. More specifically, no studies have investigated the effect of medication titration in NCC, but it has been hypothesised that medication titration could cause this effect.^7^

To address this gap in knowledge, we investigated the process of medication titration in the treatment of LDL-C in NCC. We used data from the Randomised Evaluation of Secondary Prevention by Outpatient Nurse Specialists  (RESPONSE) trial (see below). As the lifestyle risk factors were comparable in both groups in the study, the previously reported improvement of the proportion of patients on target for LDL-C in the NCC intervention group could not be explained by lifestyle changes. Additionally, participating nurses in this trial reported that the NCC intervention allowed them more frequent contact with patients and the opportunity to monitor targets more carefully.^8^ We therefore hypothesised that lipid-lowering medication titration activities occurred more often in the NCC than usual care group, and that this led to better achievement of LDL-C targets.

## Methods

### Study design and population

We used data from the RESPONSE trial, a multicentre randomised clinical trial including 754 patients from 11 centres in the Netherlands.^9^ The study was designed to quantify the impact of a practical, hospital-based nurse-coordinated prevention programme on cardiovascular risk in patients discharged after an acute coronary syndrome (ACS), as compared with usual care alone. Patients aged 18–80 years were eligible if they had been diagnosed with ACS within 8 weeks prior to entry into the trial. Patients were excluded if they (1) were unable to visit the nurse-coordinated prevention programme, (2) were not available for follow-up, (3) had a limited life expectancy (<2 years), and (4) were diagnosed with heart failure New York Heart Association class III or class IV.

### Nurse-coordinated care

Nurses participating in the NCC programme were registered nurses with at least a 4 years bachelor’s degree in nursing. They had experience in cardiovascular care and were trained in motivational interviewing. Patients in the NCC group visited the outpatient clinic up to four times during the first 6 months after inclusion, in addition to outpatient clinic visits to their cardiologist (usual care). During each nurse visit, cardiovascular risk factors were assessed, lipid profiles (including LDL-C) were reviewed, medication therapy evaluated and patient compliance with medical treatment and lifestyle recommendations was encouraged. To achieve the target lipid levels, the nurses were also encouraged to titrate medication in collaboration with the treating cardiologist.

### Data collection

Data on clinical and demographic characteristics and CHD risk factors were collected at baseline and at 6 and 12 months after randomisation. Baseline measurements were performed within 8 weeks after ACS. Patients were enrolled at an average of 4 weeks (SD 2.7) after the ACS. Data on medication use was collected at baseline, 6 months and 12 months follow-up. The data on lipid-lowering medication included number of lipid-lowering medications and, for each medication, the generic name, dosage and frequency. When LDL-C was not on target during the four NCC visits, nurses documented when medication was changed during the NCC visit, and if the treating specialists were consulted and/or patients were referred to treating specialists. All venous blood measurements were taken after a minimum of 8 hours of fasting. The target for LDL-C level was <2.5 mmol/L, as recommended by the national CVD prevention guideline at that time.^10^ Dyslipidaemia was defined by the following criteria: a history of deviated serum cholesterol values (LDL-C >4 mmol/L, HDL-cholesterol <1.0 mmol/L, triglycerides >2 mmol/L or total cholesterol >5 mmol/L) or treatment for dyslipidaemia. Further details on the trial have been published previously.^9 11^

### Lipid-lowering medication intensity and titration

Our main outcome of interest was the proportion of patients with up-titration or down-titration activities in the NCC compared with usual care, assessed by changes in lipid-lowering medication intensity at 6 months and 12 months, relative to baseline medication intensity. The 6 months follow-up visit was performed directly after completion of the NCC intervention (ie, after up to four NCC visits), while between 6 and 12 months follow-up, no specific interventions took place in either group. To account for the use of different lipid-lowering agents and dosages, the intensity of each prescription was expressed as a potential average lipid-lowering potency (ALLP, % LDL-C lowering) ranging from 13 to 70.^12^ ALLP and up-titration or down-titration was measured at 6 and 12 months follow-up. Up-titration was defined as an increase in ALLP as compared with baseline ALLP, whereas down-titration was defined as a decrease in ALLP.

As the Dutch guideline for cardiovascular risk management recommends starting with simvastatin 40 mg daily when patients are diagnosed with ACS,^13^ we defined simvastatin 40 mg as the lowest recommended dose approved for the management of ACS.

### Statistical analysis

**Figure 1:**
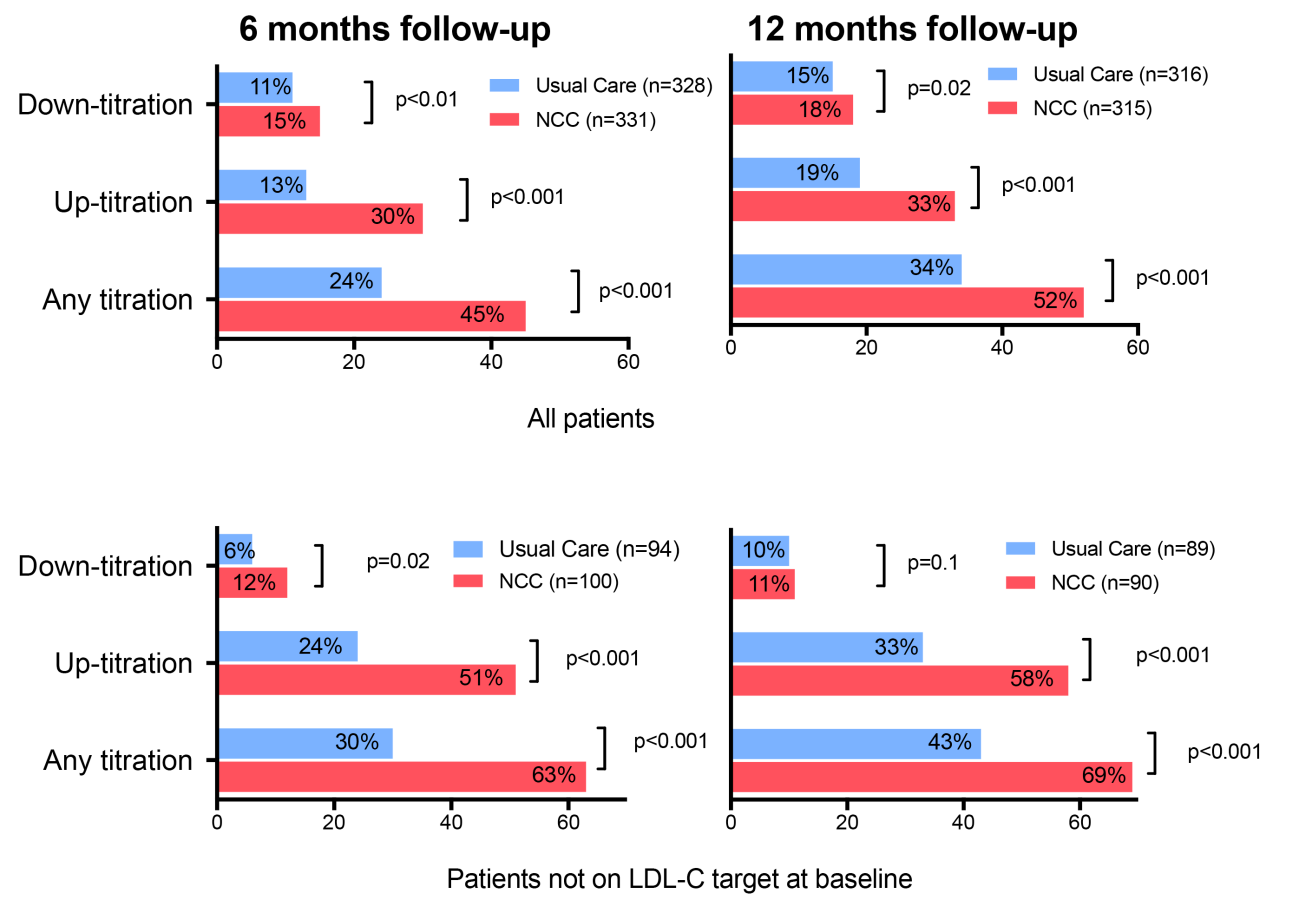
Titration activities from baseline up to 6 and 12 months follow-up in nurse-coordinated care (NCC) versus usual care patients. X-as: patients (percentage), Y-as: titration activities. Up-titration and down titrations are relative to baseline. Percentages are % of total population (upper panel) and % of population not on target (lower panel). All p values are calculated with the relevant parameter (down-titration, up-titration or any titration) versus no titration (none). Upper panel: percentage of patients with titrations of total population. All patients: Usual care at 6 months n=328, NCC at 6 months n=331, usual care at 12 months n=316, NCC at 12 months n=315. Lower panel: percentage of patients with titrations of patient population not on low-density lipoprotein-cholesterol (LDL-C) target at baseline: Usual care at 6 months n=94, NCC at 6 months n=100; usual care at 12 months n=89, NCC at 12 months n=90. Not on target is defined as LDL-C >2.5 mmol/L. Analysis applied for patients on lipid-lowering medication and patients with complete medication data.

Comparisons between groups were performed using χ^2^ test for categorical variables. Differences between characteristics of up-titrated and down-titrated patients were analysed by the χ^2^ test. The p values presented in figure 1 were up-titration versus no titration (none), and down-titration versus no titration (none). A two-sided p value of <0.05 was considered statistically significant. As ALLP is not a continuous variable, we expressed ALLP as a sum of the prescribed potencies per group. SPSS Statistics for Macintosh, V.22.0. (Armonk, New York, USA) was used for descriptive statistical analyses.

In order to include the NCC intervention effect at 6 months, we plotted ALLP changes between baseline and 6 months. We assessed if patients in the NCC group who were (not) on target at baseline received greater intensity changes than those in the usual care group by estimating the interaction between treatment arm and (not) being on target at baseline in a linear regression analysis. These analyses were performed using Stata V.13.1 (College Station, Texas, USA).

To check for selective dropout, we used a logistic regression model and regressed a binary variable indicating missingness (1=yes, 0=no) on the following variables as predictors of missingness under the hypothesis that if all ORs were close to 1, selective dropout due to these predictors is unlikely: age, gender, education level, index event, history of CVD, alcohol, smoking at baseline, diabetes mellitus and their interaction with randomisation group.

## Results

Our population consisted of 754 patients with a mean age of 58 years (SD 10.1), 80% were men. The majority (73%) had no history of CVD prior to the index hospitalisation. As previously described, baseline patient characteristics did not differ between the NCC and usual care groups.^9^ In the NCC group, 92% of 365 patients attended all four NCC consultations as scheduled during the first 6 months. In total, 46 patients in the intervention and 33 patients in the usual care group had one or more missing values for our analyses (11%). Logistic regression did not reveal an indication for selective dropout between the NCC and usual care group.

**Table 1:** Low-density lipoprotein-cholesterol (LDL-C) and the average lipid-lowering potency (ALLP) in nurse-coordinated care (NCC) versus usual care patients at baseline and 6 and 12 months follow-up

	Baseline*		F6			F12		
Parameter	NCC (n=365)	Usual care (n=367)	NCC (n=356)	Usual care (n=346)	p Value†	NCC (n=357)	Usual care (n=352)	p Value†
On lipid-lowering medication, n (%)	350 (96%)	352 (96%)	345 (96%)	335 (96%)	0.70	331 (93%)	328 (94%)	0.64
LDL-C OT (≤2.5 mmol/L)	247 (68%)	249 (68%)	284 (80%)	241 (69%)	<0.001	263 (74%)	223 (64%)	<0.01
Total ALLP‡ (% LDL-C lowering)	14.366	13.943	15.003	14.030	NA	14.564	13.964	NA

*At baseline differences not statistically significant at the 5% level.

†Calculated between NCC and usual care (between-groups).

‡ALLP: the ALLP as an indicator of lipid-lowering medication intensity using the method by Besseling *et al* 12 (ref).

Total ALLP is the sum of the prescribed lipid-lowering potencies (%) per group.

NA, not applicable; OT, on target.

### Titration activity outcome

The proportion of patients with up-titration or down-titration of lipid-lowering medication from baseline to 6 and 12 months follow-up was higher in the NCC group as compared with the usual care group (figure 1). Reflective of the NCC titration intervention, markedly more lipid-lowering titration was seen at 6 months follow-up in the NCC group compared with the usual care group (any titration in all patients 45% vs 24%, p<0.001) (figure 1). At 12 months, a slight increase of titration activities was seen in both groups, yet a statistically significant difference between the two groups remained (52% vs 34%, p<0.001). While both up-titration and down titration in ALLP were seen in both groups, more patients in the NCC than in the usual care group were up-titrated (6 months 30% vs 13%, p<0.001; 12 months 33% vs 19% p<0.001).

**Figure 2:**
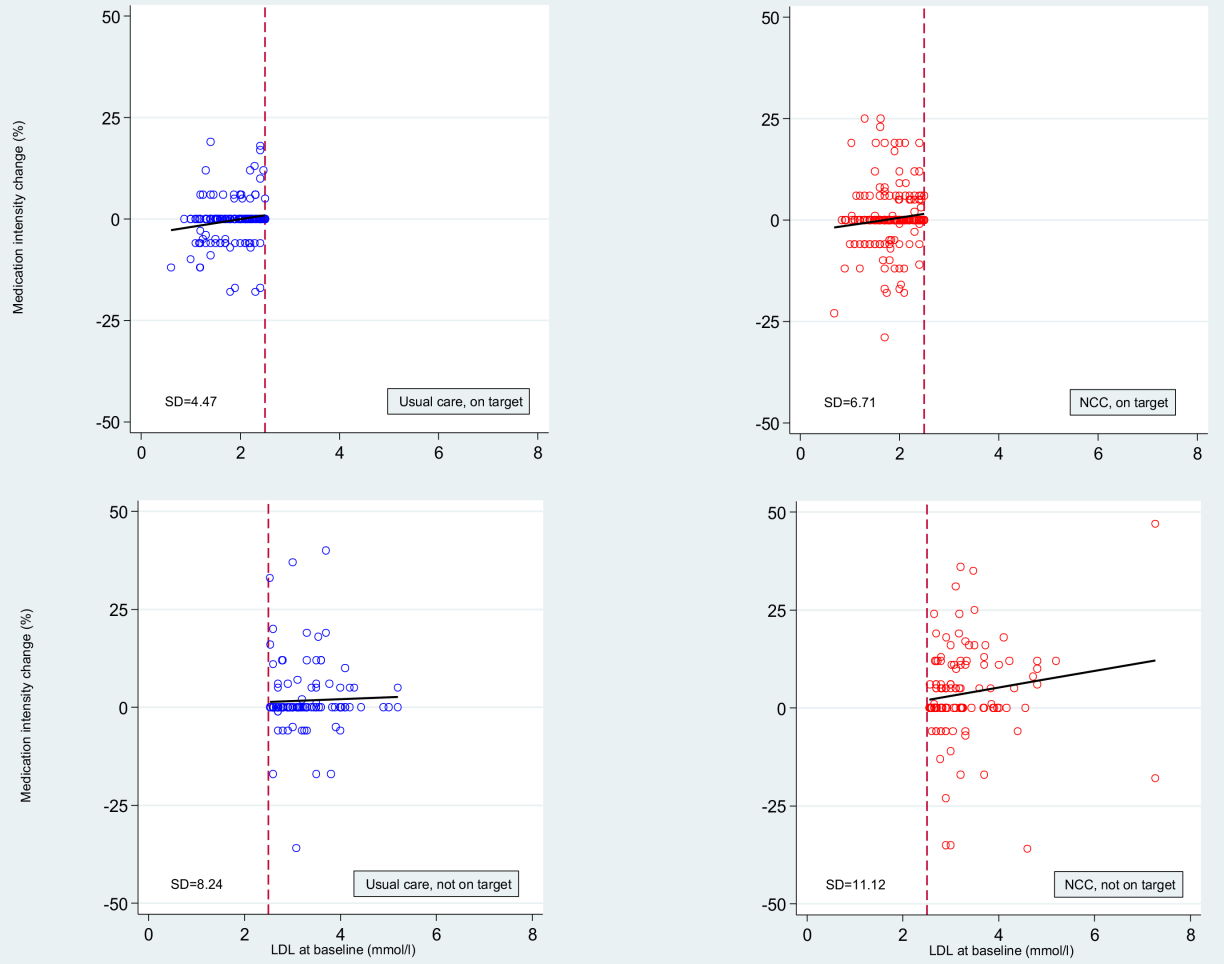
Medication intensity (ALLP) changes between baseline and 6 months, by (not) being low-density lipoprotein-cholesterol (LDL-C) target at baseline for nurse-coordinated care (NCC) (red dots) and usual care (blue dots) patients. Dots represent individual patients. The right lower graph shows, on average, more medication intensity changes in NCC patients not on target at baseline compared with usual care patients (left). The red dashed vertical lines indicate the cut-off LDL-C serum concentration of 2.5 mmol/L. The black lines are the slopes based on a linear regression analysis of the medication intensity changes against LDL-C levels at baseline. ALLP, the average lipid-lowering potency (ALLP, % LDL-C lowering) as an indicator of lipid-lowering medication intensity; LDL, low-density lipoprotein.

In patients not on LDL-C targets at baseline (figure 1), most titration activities (up or down) and the largest difference between NCC and usual care groups were observed in the first 6 months (6 months: 63% vs 30%, p<0.001; 12 months: 69% vs 43%, p<0.001). Similarly, in patients not on target at baseline, also up-titration activities were more often observed in the NCC than in the usual care group, particularly in the first 6 months (6 months: 51% vs 24%, p<0.001; 12 months: 58% vs 33%, p<0.001).

figure 2 shows all ALLP changes between baseline and 6 months as a function of LDL-C at baseline for NCC and usual care patients (not) on target at baseline. On average, NCC had an (absolute) effect on ALLP compared with usual care alone, especially if patients were not on target at baseline (slope 2.3, (95% –0.11 – 4.72)). The differences in SD between NCC and usual care reaffirm the spread of ALLP between these two groups.

### Lipid-lowering medication data

At baseline, the proportion of patients on lipid-lowering medication was high in both the NCC (96%) and the usual care group (96%), and 68% of all patients were on LDL-C target at baseline (table 1). Simvastatin (43%), followed by atorvastatin (41%), were the most commonly used lipid-lowering medications prescribed at baseline. During follow-up, a higher proportion of patients in the NCC group were on target compared with the usual care group (6 months: 80% vs 69%, p<0.001; 12 months: 74% vs 64%, p<0.01). Total ALLP was slightly higher in the NCC as compared with usual care at both 6 months (15.003 vs 14.030) and 12 months (14.564 vs 13.964) (table 1).

### Characteristics of up- titrated  and down-titrated patients compared with patients with no titration

There were no differences in demographic or clinical characteristics as age, gender, level of education, index event or cardiovascular risk factors of up-titrated and down-titrated patients (data not shown). However, up-titrated patients had dyslipidaemia more frequently as compared with patients with no titration (79% vs 70%, respectively, p=0.04), and up-titrations were associated with allocation to the NCC group (62% vs 43%, p<0.001).

Down-titrated patients had dyslipidaemia less frequently as compared with patients with no titration (56% vs 70%, respectively, p=0.02). Down-titration was also more frequently seen in patients allocated to the NCC group as compared with patients with no titration (55% vs 43%, respectively, p=0.02).

## Discussion

Our study demonstrates that NCC in patients with ACS is associated with more frequent lipid-lowering medication titration and with higher ALLP values to reach LDL-C targets as compared with usual care alone. These titrations took place in a relatively short amount of time (four visits in 6 months after an ACS), but changes made in the first 6 months in lipid-lowering medication were also observed 6 months after completion of the NCC programme, and were reflected in a higher proportion of patients reaching targets for LDL-C. Our study took place in a context of high prescription rates of lipid-lowering medication (96% in both groups at baseline). Despite these high prescription rates, the target for LDL-C (2.5 mmol/L) was not reached in a considerable number of patients in both groups (NCC 26% vs usual care 36%). Our study shows that there is considerable room for individual tailoring of lipid-lowering medication therapy, with more both up-titration and down-titration in medication intensity in the NCC group. While lifestyle modification could account for some changes in LDL-C levels, it is unlikely that this can explain the differences in the higher proportion of patients on target in the NCC group, as lifestyle risk factors were comparable through the study up until 12 months follow-up.^9^ Despite a small difference in the total sum of ALLP in both groups at 6 and 12 months, the proportion of individuals on target for LDL-C was markedly higher in the NCC group as compared with the usual care group, reflecting the efficacy of adequate individual medication titration.

Large proportions of high-risk cardiovascular patients have been shown to discontinue their statin therapy, emphasising the need for healthcare providers to discuss medication use with their patients.^14^ An integral part of the NCC intervention in our study was interviewing patients about their compliance, asking about barriers concerning adherence and titrating medication (ie, lipid lowering medication) to optimise adherence. Our data showed that down-titrations were made in NCC patients. A possible reason for these down-titrations could be maintaining compliance in case of side effects, as patients on high-intensity statin therapy who experience side effects (such as myopathy) are likely to be less compliant than patients down-titrated to a better tolerated statin intensity.

According to the European Society of Cardiology (ESC) guideline, reducing dosage is an effective approach for enhancing medication adherence.^1 15^ Nurse-coordinated programmes are associated with modest but positive effects on reducing cholesterol levels according to recent meta-analyses.^6 16^ However, studies assessing patients’ medication adherence found improved patient adherence in one study^17^ and no differences between NCC and usual care in two other studies.^18 19^ Reasons for poor patient adherence are multifactorial. According to the WHO, reasons for medication non-adherence are categorised in five groups: health system, condition, patient, therapy and socioeconomic factors.^15^ In particular, education and frequent follow-up visits have been shown to be associated with improved adherence,^20^ and NCC potentially positively influences several of these categories. While we found that targets for LDL-C were more frequently achieved in NCC, more research on the role of NCC to improve medication adherence in general would be valuable.

Patients allocated to the NCC group reached the target level of LDL-C in a short period of time after discharge. This is likely to be beneficial, as several trials have demonstrated important reductions in major cardiovascular events from lowering cholesterol, especially LDL-C.^21^ The total sum of ALLP for NCC patients was only slightly higher compared with patients in usual care. This should be seen as clinically relevant as this difference probably led to a larger proportion of patients achieving target level for LDL-C, and the clinical benefits of LDL-C lowering in general are well known.^22 23^

Secondary prevention based on nurses’ collaboration has the potential to improve patient care. While healthcare organisations differ widely across Europe, the ESC prevention guidelines recommend a multidisciplinary team for secondary prevention including physicians and nurses. In some countries, secondary prevention is mainly the task of physicians, while in others, specially educated and trained nurses play a more prominent role.^1^ Physicians and nurses are recommended to work together as a team to provide the most effective multidisciplinary care. NCC has proven to be effective in reducing risk factors,^6 9^ anxiety and depression,^24^ and nurses reported to appreciate participating in such multidisciplinary teams.^8^ Therefore, depending on local practice, integrating NCC should be considered in secondary prevention in ACS patients.

### New developments and limitations

The ESC guideline target for LDL-C changed from 2.5 mmol/L to 1.8 mmol/L after the completion of inclusion of patients in the RESPONSE trial.^15^ This change increases the need for new initiatives to reach LDL-C targets in patients with CHD, as it is shown that only a minority of patients reach these stricter targets.^4^ The specific role of NCC in this process needs further evaluation, especially with the upcoming availability of new pharmacological strategies, such as PCSK9-inhibitors.

Moreover, it should be noted that our data on medication use were based on self-report by professionals, and not corroborated with additional questionnaires regarding adherence or pill counts. While side effects were discussed with patients during NCC consultation, we did not specifically collect data on such side effects. This might be a valuable part of follow-up research. Furthermore, we did not correct for possible confounders such as lifestyle factors in our analysis. The development of a model with the hypothesised pathways between LDL-C on target and NCC interventions, including all potential confounders of this relation, could potentially help to more fully investigate the association between NCC titration and LDL-C on target. Such causal mediation analysis may be used to investigate the causal role of titration activities relative to other factors associated with NCC in future trials.^25 26^

## Conclusion

In conclusion, among patients hospitalised for ACS, NCC resulted in more intensive medication titration compared with usual care alone. The greater proportion of patients on LDL-C target at 6 and 12 months follow-up is likely explained by the more intensive titration of lipid-lowering medication in NCC patients compared with usual care alone. Merely starting the guideline-recommended dose is insufficient to reach the guideline-recommended LDL-C target level. NCC, combined with guideline-based titration recommendations, can improve ACS patient outcomes and should become part of routine daily practice.
